# Palm Oil in Myanmar: A Spatiotemporal Analysis of the Effects of Industrial Farming on Biodiversity Loss

**DOI:** 10.9745/GHSP-D-17-00132

**Published:** 2018-03-21

**Authors:** Khristopher Nicholas, Jessica Fanzo, Kytt MacManus

**Affiliations:** a The Earth Institute, Columbia University, New York, NY, USA.; b Institute of Human Nutrition, Columbia University Medical Center, New York, NY, USA. Now with Johns Hopkins Berman Institute of Bioethics, Baltimore, MD, USA.; c Center for International Earth Science Information Network, Earth Institute, Columbia University, Palisades, NY, USA.

## Abstract

**Background:**

Palm oil consumption is potentially deleterious to human health, and its production has resulted in 11 million hectares of deforestation globally. Importing roughly 394,000 metric tons of palm oil in 2012 alone, the Burmese government has recently pushed for intensive oil palm development to sate domestic demand for consumption and become international market players. Given well-studied linkages between biodiversity loss and ecosystem instability, this study aims to characterize the nature of deforestation for oil palm production in Myanmar, its relationship to increased biodiversity loss, and contextualize the potential impacts of this loss on diets and human health in rural Myanmar.

**Methods:**

First, a GIS land suitability analysis overlaying spatial data on rainfall, elevation, and slope was conducted in order to identify areas of Myanmar best suited to oil palm tree growth. Second, after narrowing the geographic range, vegetation indices using varying spectral band models in ENVI (Environment for Visualizing Images) allowed a more granular examination of changes in vegetation phenology from 1975 to 2015. Lastly, ground truthing permitted an in-person verification of GIS and ENVI results and provided contextual understanding of oil palm development in Myanmar.

**Results:**

GIS analysis revealed that the Tanintharyi Region, one of the most biodiverse regions in Myanmar, is highly suitable for oil palm growth. Next, vegetation indices revealed a progressive shift from smallholder farming, with little observable deforestation between 1975 and 1990, to industrial oil palm plantations all throughout Tanintharyi starting around 2000—a shift concomitant with biodiversity loss of primary forestland. Ground truthing indicated that plantation development has advanced rapidly, though not without barriers to growth.

**Conclusions:**

If these trends of Burmese oil palm intensification continue, 4 key outcomes may follow: (1) even higher levels of biodiversity loss, (2) increased access and affordability of edible palm oil, (3) decreased importing of palm oil, and (4) large profits made from selling excess palm oil on the international market. Although the first 2 outcomes may adversely affect low-income Burmese populations, the latter 2 may bode well for the domestic economy and international trade partners, thus encouraging competing interests. This increased domestic access and affordability of palm oil may increase consumption and cause increased prevalence of cardiovascular disease, diabetes, and obesity. Finally, this biodiversity loss concurrent with industrial deforestation may disproportionately impact vulnerable, rural communities.

## Abstract (precis)

Satellite imagery analysis reveals a progressive shift from smallholder farming to industrial oil palm plantations in rural Myanmar, concomitant with biodiversity loss. Although industrial palm oil cultivation may help the local economy flourish, rural communities assume the dual burden of ecosystem instability from deforestation and potential health risks from increased palm oil consumption.

## INTRODUCTION

Palm oil is a $50 billion global market and is used in approximately 50% of products in European supermarkets.^1^ From agrochemical and food production to biofuels and cosmetics, palm oil is used as a key ingredient across many industries.

There have been many studies that demonstrate the potentially deleterious health outcomes when palm oil is used as cooking oil. In a landmark 1999 study on the influence of palm oil consumption on health, researchers Ebong et al. of the University of Calabar in Nigeria demonstrated while palm oil in the fresh (uncooked) state is rich in antioxidant vitamin A precursors such as beta-carotene and vitamin E, once it reacts with oxygen during the cooking process, saturated fatty acids are introduced and the plasma lipid profile becomes adverse—specifically, low-density lipoprotein (LDL) (“bad”) cholesterol levels increase. Both of these changes pose significant threats to physiological and biochemical functions.^2^^–^^4^ Furthermore, the malignancy of these effects is compounded each time the oil is reheated when used for cooking. Some low-income families may reuse palm oil when cooking household meals, as do restaurants or street vendors in commercial food settings.^5^ These health consequences could be significant, especially as cardiovascular disease, obesity, and diabetes increase in prevalence in low- and middle-income countries. As this trend progresses, the need for healthier and sustainably sourced fats will become even more pressing.

There are potentially deleterious health outcomes when palm oil is used as cooking oil.

Southeast Asia presently supplies the vast majority of palm oil globally.^6^^,^^7^ Of the 53.8 million tons of palm oil produced globally in 2012, Indonesia and Malaysia alone comprised 89% of total production.^6^ As a large-scale palm oil supplier, Myanmar is young compared with Indonesia and Malaysia. Until recently, Myanmar was a palm oil net importer. According to the Myanmar Edible Oil Distribution Association, in 2012 alone, Myanmar imported roughly 394,000 metric tons of palm oil, primarily from Indonesia and Malaysia, at a cost of $376 million.^8^ In a survey of 900 households on edible oil preferences among groundnut, sesame, and palm oil, Han et al. found that 96% of households ranked palm oil as the most affordable edible oil while 99% ranked palm oil as least suitable to health.^8^ Similar findings that palm oil is the cheapest and most readily available edible oil in Myanmar have been noted by S. Downs, PhD (written communication, January 2018).

To satisfy domestic demand and mitigate the import of palm oil, the Burmese government has rapidly pushed for intensive palm oil production—what it refers to as an “edible oil self-sufficiency policy.”^9^ In 2014, for example, there were over 140,000 hectares of oil palm plantations in Myanmar with an additional 400,000 allocated for future development to more than 40 local companies and 3 international companies.^10^ Accordingly, in Myanmar, palm oil consumption is catalyzed by its affordability and availability while the prospects for its production, at least in the near future, appear to be safeguarded by a government invested in ensuring edible oil self-sufficiency.

To satisfy domestic demand and mitigate the import of palm oil, the Burmese government has instituted an “edible oil self-sufficiency policy.”

There are numerous studies focusing on palm oil industries in Indonesia and Malaysia and their historical land-use patterns,^11^^–^^13^ but few have focused on Myanmar's palm oil production. This article analyzes spatiotemporal trends in land use from 1975 to 2015 in rural Myanmar, focusing on oil palm cultivation, and its effects on land biodiversity. We start first by providing an overview of the impact of industrial farming on planetary and human health to provide background context on the importance of this issue.

## CONTEMPORARY FOOD SYSTEMS: THE IMPACT OF INDUSTRIAL FARMING

The push for oil palm cultivation is significant because of its role within a global food market. When industrial food production began replacing smallholder farming in the 18th and 19th centuries, the primary objective was to meet rising food demand.^14^ This agricultural revolution paved the way for the dawn of synthetic agrochemicals and the global distribution of high-yield seeds.^15^ Unintentionally, this movement also helped spawn today's complex global food system—a system in which industrial oil palm cultivation has environmental, nutritional, and ethical implications. Intensive agriculture affects ecosystems by the use and release of nitrates and pesticides and converts natural ecosystems to industrial farmland.^15^ This conversion is strongly linked with the degradation of key ecosystem services and accelerates soil nutrient depletion. Furthermore, as mass food production grew easier, it became cheaper to export high-energy, nutrient-poor foods in bulk.

According to the Intergovernmental Panel on Climate Change, agriculture contributes between 10% and 12% of global greenhouse gas emissions.^16^ Industrial farming occupies enormous swaths of land, makes extensive use of agrochemicals, reduces (or eliminates) fallow periods that allow natural soil nutrient regeneration, and heavily utilizes high-yield seed variety.^17^ The disadvantages of this type of farming can be divided into two categories with considerable overlap: planetary health and human health.

Industrial farming can have negative impacts on both planetary health and human health.

### Planetary Health and the Importance of Biodiversity

Humans derive considerable benefit from natural ecosystems. Natural resources including lumber, fossil fuels, and food are the most obvious. However, there are economic and ecological benefits that are not readily quantifiable. For example, primary forests can reduce flood damage, absorb large amounts of atmospheric carbon dioxide, and purify water.^17^ The benefits gleaned from these services can be drastically reduced by anthropogenic environmental degradation. The focus of this article and one of the most damaging of these degradations is biodiversity loss.

On a macro scale, ecosystem stability depends on the “diversity of form and function of the constituent species.”^18^ This stability is often measured by species density, or biodiversity, of fauna and flora within a given area. More specifically, the importance of biodiversity in tree species is extremely important. Forests and their soils act as a crucial sink for anthropogenic carbon emissions, storing roughly 45% of terrestrial carbon.^19^ Yet as of 2013, agricultural deforestation alone resulted in the loss of 45% of temperate deciduous forests and 27% of tropical rainforests globally.^19^ In their 2013 study, Gamfeldt et al. studied the effect of biodiversity of tree species on 6 ecosystem services. Significantly, this study found that forests with greater tree diversity produced 54% more biomass, captured 11% more carbon in the soil, and boasted 31% more diversity of understory plant species.^19^ Furthermore, the link between biodiversity loss and negative human health outcomes is most keen in vulnerable populations.^20^ Primary and diverse forestland provides not only lumber and cooking fuel in these communities but also protection against storms, medicinal ingredients, seasonal fruits and vegetables, habitat for animals (a key protein source), and a cultural landmark.^20^ As industrial monoculture agriculture progresses, the availability of these local resources may decrease, leading to measureable impacts on diet composition. In Myanmar, the Coastal zone (including the Tanintharyi Region, the site of current and future oil palm plantation development) has the highest poverty rates in the country at 53.1%.^21^ As such, the human health impacts of biodiversity loss from deforestation may be most acutely felt by these vulnerable populations in surrounding communities within Tanintharyi.

It is clear that the maintenance of biodiversity is paramount for planetary health and the ecosystem services that improve human health, particularly in vulnerable communities. It is also clear that industrial monoculture agriculture greatly reduces biodiversity via deforestation. Therefore, the large, highly simplified industrial monocultures that are phasing out smallholder farming systems in the Global South are bad not just for planetary health but also human health. For example, a diet comprised of only a few recurring food groups is less healthful than a diet of diverse foods from different food groups.^22^ Food homogeneity has increased by 16.7% globally over the past 50 years with many East and Southeast Asian countries showing an in-country homogeneity increase of 35.7%.^23^ In other words, these countries have witnessed lower agricultural and diet diversity due in part to the intensification of large-scale industrial monocultures such as oil palm. In fact, over the past 50 years, the rate at which palm oil consumption has grown in national food supplies and the rate at which its cultivation is spreading to new lands is among the highest of any crop in the world.^23^

Industrial monoculture agriculture greatly reduces biodiversity via deforestation.

With recent global climate concerns, agriculture intensification, urbanization, and shifted cultural valuations toward environmentally costly products, global efforts to combat hunger can no longer focus solely on maximizing production with industrial monoculture agriculture. Instead, these factors highlight the need to produce food that is sustainably grown, culturally acceptable, affordable, readily accessible, and nutritious.^24^^,^^25^ The “Sustainable Diet” approach values each of these important considerations.

### Human Health and the Sustainable Diets Framework

**FIGURE 1.**
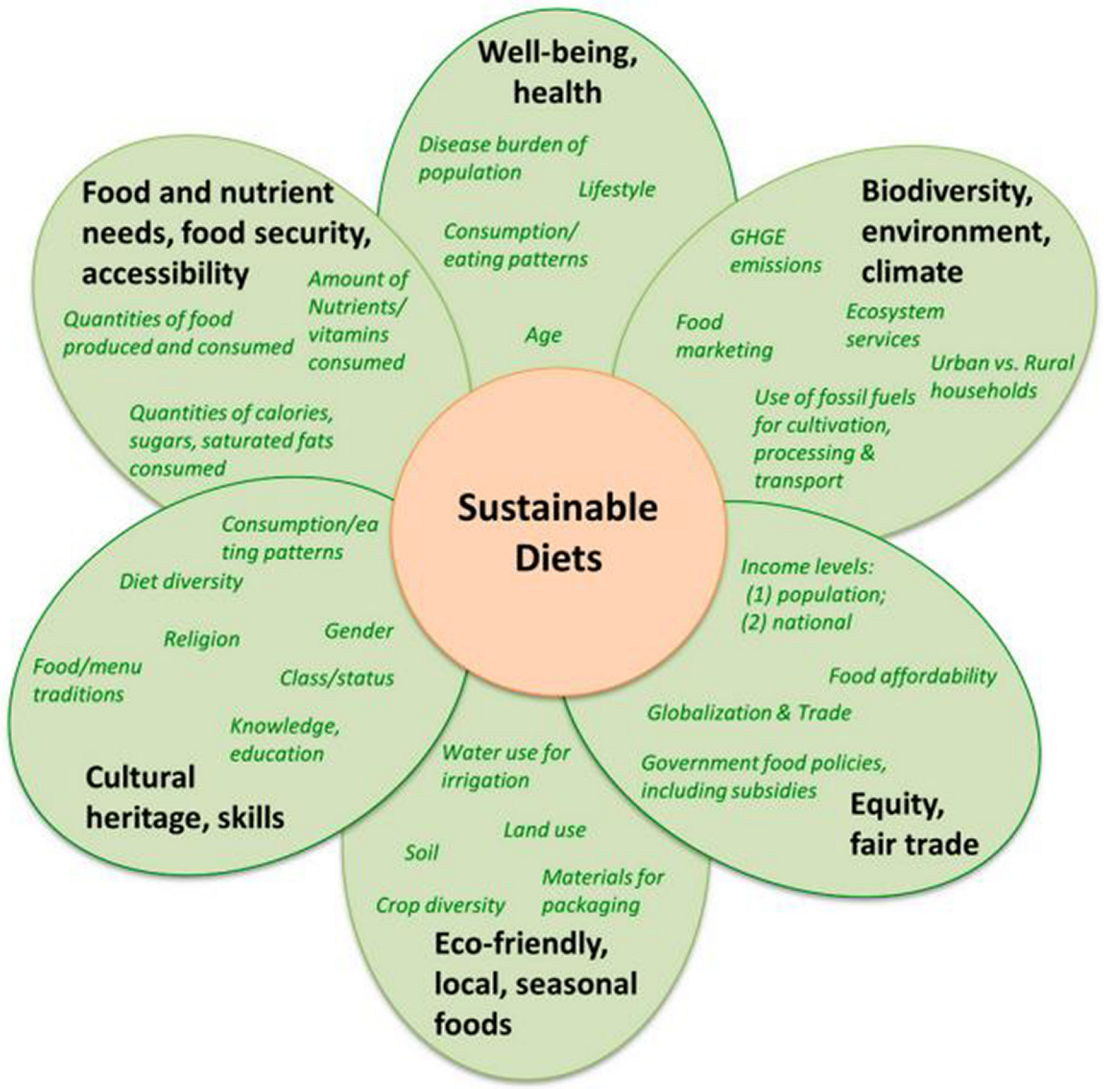
Components of the Sustainable Diet Abbreviation: GHGE, greenhouse gas emissions. Source: Adapted from Johnston, Fanzo, and Cogill (2014).^24^

Burlingame (2012) defined sustainable diets as “those diets with low environmental impacts which contribute to food and nutrition security and to healthy life for present and future generations.”^26^^(p7)^ Accordingly, the 6 key interconnected determinants of a sustainable diet are: (1) well-being, health; (2) biodiversity, environment, climate; (3) equity, fair trade; (4) eco-friendly, local, seasonal foods; (5) cultural heritage, skills; and (6) food and nutrient needs, food security, accessibility (Figure 1).^24^

Any fluctuation of one of the key determinants will have a corresponding effect on the quality of the sustainable diet. For example, under the “biodiversity, environment, climate” determinant, if greenhouse gas emissions increase from intensive slash-and-burn agriculture, then the sustainable diet will be decidedly less sustainable. Each component is also inextricably linked to the other. With an increase in greenhouse gas emissions, for example, the disease burden of a population process might increase the prevalence of respiratory diseases because of soot and ash released from the burning of hydrocarbons, and so on.

To analyze sustainable diets across countries, researchers have developed a suite of 10 principles that seek to integrate elements of nutrition and the environment in food-system analyses (Table 1). This study focuses on the third principle, maximizing landscape biodiversity. As discussed, biodiversity loss has implications on ecosystem services, food production, and human health. This underscores the link between planetary and human health. That is, if palm oil becomes even more affordable and readily available under the government's self-sufficiency push, lower-income communities in Myanmar may continue to consume it in equally abundant amounts or even increase their consumption of palm oil. Importantly, of these lower-income communities, those located in Tanintharyi Region may additionally bear health burdens induced by biodiversity loss from deforestation.

**TABLE 1.** Suite of 10 Principles Integrating Nutrition and the Environment in Food Systems

Principle	Example(s)
1.Be coherent with health-based dietary guidelines	Nutrient intake compared with age-, gender-, and health condition-based adequacy
2.Maximize nutritional output per unit of input (energy, land, water, nutrients, labor) in production, post-harvest management, and processing	Nutrients produced per unit of input. High-quality seeds, life cycle analyses, and agricultural subsidies help maximize this metric
3.Maximize biological diversity at different levels of the food system (in the landscape, the markets, and the diets)	Investment in local seed banks, changing consumer behavior, shared market space, and landscape preservation policies
4.Minimize greenhouse gas emissions	Promote balanced meat consumption
5.Minimize chemical pollution and water contamination	Responsible use of fertilizers and pesticides, farmer training programs
6.Minimize waste and enhance recycling of nutrients throughout the food system	Percentage of food wasted along value chain
7.Maximize food safety	Estimated risk for food contamination
8.Ensure human rights, including rights to food and health, of food system workers are supported	Fair hours and wages; health risk exposure; tradeoffs between jobs created and divisions along socioeconomic lines
9.Improve equity and affordability of healthy food items	Minimize cost of nutritious diets for low-income consumers
10.Be adapted to local and changing conditions	Culturally relevant and acceptable diets

Source: Remans et al. (2015).^27^

Biodiversity loss has implications on ecosystem services, food production, and human health.

## METHODS

Our approach to analyzing land-use changes in Myanmar used 3 methods with increasing geographic specificity: (1) Beginning with a land suitability analysis using geographic information systems (GIS) modeling, we identified regions of interest for oil palm cultivation. (2) We then analyzed these regions using Exelis' ENVI (Environment for Visualizing Images) satellite image processing. (3) Finally, we traveled to these regions for photo-based observations and ground truthing (verification of remote sensing models).

### GIS Modeling: Land Suitability Analysis

GIS modeling allows the user to spatially relate different types of data, cartographically display these relationships, and query the data to identify new patterns in these relationships. First, we conducted a land suitability analysis for oil palm cultivation in Myanmar. That is, we analyzed Myanmar to identify which regions are conducive to oil palm cultivation based on rainfall, elevation, and slope parameters. This land suitability analysis is based on a simple assumption: above or below certain thresholds, oil palm trees will not grow. These climate data are freely available online through databases such as WorldClim and the Myanmar Information Management Unity.

We first conducted a land suitability analysis to identify which regions in Myanmar are conducive to oil palm cultivation based on rainfall, elevation, and slope parameters.

First, it was necessary to prime the data. With most natural phenomenon (e.g., elevation), data come referenced in preset groups. We had to adapt these data to our purposes. With all 3 natural parameters (elevation, rainfall, slope), we created a custom symbology using a binary system in which color ‘A’ represented unsuitable land while color ‘B’ represented suitable land. Suitability levels were based on land with rainfall between 2,400–3,200 mm/year, on a slope of <16%, and at an elevation of <400 m and <100 m (see the supplement for details on selecting parameter thresholds). After creating these binary bins, we used the Reclassify Tool with the custom-binned file as our input. The output from this tool was visually identical to the input map, except that land with color A now refers to value ‘0’ while color B refers to value ‘1’. This step was necessary for the final overlay analysis. Note that an additional step was required for slope. Because slope is a derivation of elevation and the units and scale of elevation (z) are different than those of surface measurements (x, y), a z-factor conversion was necessary. In Myanmar, based on a 20° N latitude, we used a z-factor conversion constant of 0.00000956. Following this step, all that remained was the final overlay.

After having reclassified these datasets into maps referencing binary values 0 and 1, all 3 natural parameters had an identifiable virtual language and interacted more smoothly. Using the Weighted Overlay tool, we input the 3 natural parameter raster datasets and created a custom evaluation scale with ‘0’ as ‘NODATA’ and ‘1’ as ‘1’. The tool now had a translation code, creating a dual-colored map. Black represented the most suitable land for oil palm cultivation in Myanmar and white represented non-ideal geographies. This preliminary model construction revealed areas of agricultural interest in Myanmar that were then juxtaposed against known areas of forested landscape and high biodiversity.

### Remote Sensing Analysis: ENVI Satellite Imaging

Since the mid-1900s, Global Land Surveys (GLS) have captured terabytes of satellite imagery. Over the decades, as scientists launched new Landsat satellites into orbit, this imagery became clearer and more abundant, and it captured more information from the electromagnetic spectrum not visible to the human eye. This latter feature renders modern imagery highly useful, because advanced image processing software can manipulate the spectral bands and discover new land-use patterns. ENVI is one such software that we used.

In the chosen area of study obtained from GIS modeling, we analyzed GLS satellite imagery from 1975, 1990, 2000, 2005, 2010, and 2015. With the requisite satellite imagery imported into ENVI, we gained key insight into spatiotemporal land-use patterns over time, such as deforestation trends, the development of transportation infrastructure, and changes in relative intensity of industrial oil palm farming. For each of these images, we selectively activated certain spectral bands to produce 2 images each: (1) a pseudo-natural color image in which healthy vegetation is green, recently cleared fields are lighter, and browns and yellows indicate unhealthy vegetation, and (2) a false-color image whose properties reveal distinct characteristics about vegetation abundance. The specifications for these images are outlined in Table 2.

**TABLE 2.** Summary of Spectral Band Models Used in Remote Sensing Analysis

Spectral Representation	GLS Image Year	Description
Pseudo-Natural Color	1975, 1990, 2000, 2005, 2010, 2015	Selective activation of Landsat bands 3, 2, and 1. Ground features appear in colors similar to reality. Healthy vegetation is green, cleared lands are light, and unhealthy vegetation is brown and yellow. Roads are gray.
Vegetation	1975, 1990, 2000, 2005, 2010	Selective activation of bands 4, 5, and 1. Vegetation appears in shades of red, brown, oranges, and yellow. Soil may be green or brown. Differences in vegetation types easier to discern.
Color Infrared	2015	Selective activation of bands 5, 4, and 3. A highly detailed yet simple model to analyze vegetation. Vigorous vegetation growth is intense red, light pink is dead vegetation.

We analyzed satellite imagery from 1975 to 2015 to discern land-use patterns over time.

Having identified areas of agricultural interest based on the land suitability analysis, we further investigated the impact of oil palm plantation growth on local flora and fauna. Based on these spectral band models dating from 1975 to 2015, a more granular, detailed examination of suitable arable land was possible.

### Ground Truthing

GIS modeling and ENVI analysis provided key insights but still needed validation. Ground truthing helps provide context and validation in a complex Burmese sociopolitical backdrop. Having identified key areas of industrial deforestation, our field visit verified the extent and nature of deforestation and informally allowed us to gain insight into community relationships with palm oil industries.

## RESULTS

### GIS Modeling: Land Suitability Analysis

**FIGURE 2.**
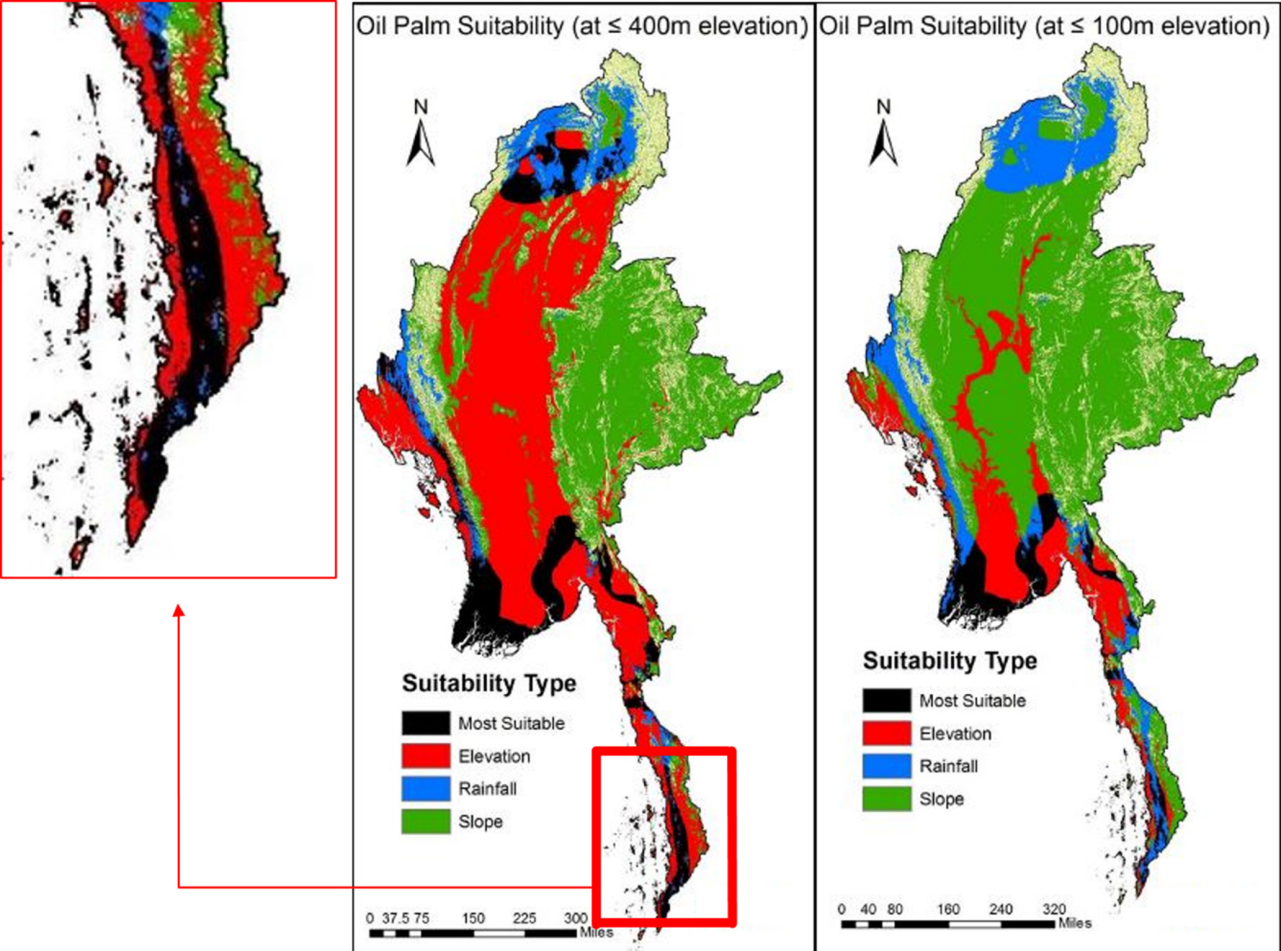
Land Suitability Analysis The most suitable area represents land with rainfall between 2,400 and 3,200 mm/year, on a slope of <16%, and at an elevation of either <400 m (left) or <100 m (right), the latter based on studies conducted in Malaysia^28^ and Myanmar,^10^ respectively. There is 38% more “most suitable” land in the 400 m versus 100 m elevation models. The expanded area is the Tanintharyi region where both this model and literature reviews confirm palm oil development will take place.

From the suitability overlay analyses, it was apparent that there were 2 regions of interest: the Irrawaddy Division comprising the southwestern peninsula and the Tanintharyi Division on the long southern strip (Figure 2).

Naturally, there is a greater proportion of most suitable land in the more generous 400 m-elevation cutoff (38% more suitable land, specifically). We identified the Tanintharyi Region, the expanded area in the map, as the region of most interest. It not only proves very suitable land for oil palm plantations in this model, but also is the most biodiverse region in Myanmar.^10^ This region also has the highest poverty rates in Myanmar. We will, however, note that many of the issues raised by oil palm cultivation in Tanintharyi outlined below may be ameliorated by the partial shifting of cultivation to the Irrawaddy Region, though of course this will bring its own set of challenges. In sum, Tanintharyi demonstrates a confluence of agricultural activity, deforestation, biodiversity, and vulnerable communities that render it indicative of similar communities around the globe and will be the focus of our analyses here.

Land suitability analysis identified Tanintharyi Region as the region of most interest for oil palm cultivation.

### Remote Sensing Analysis: ENVI Satellite Imaging

**FIGURE 3.**
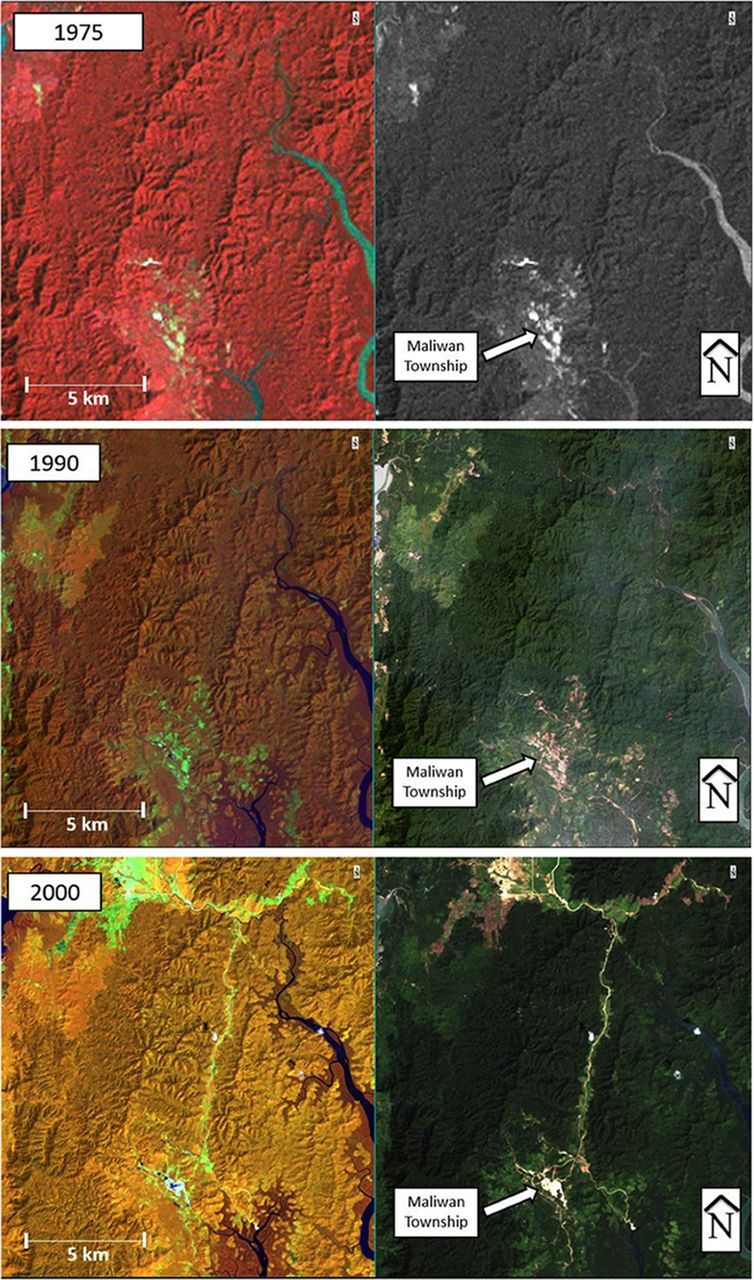

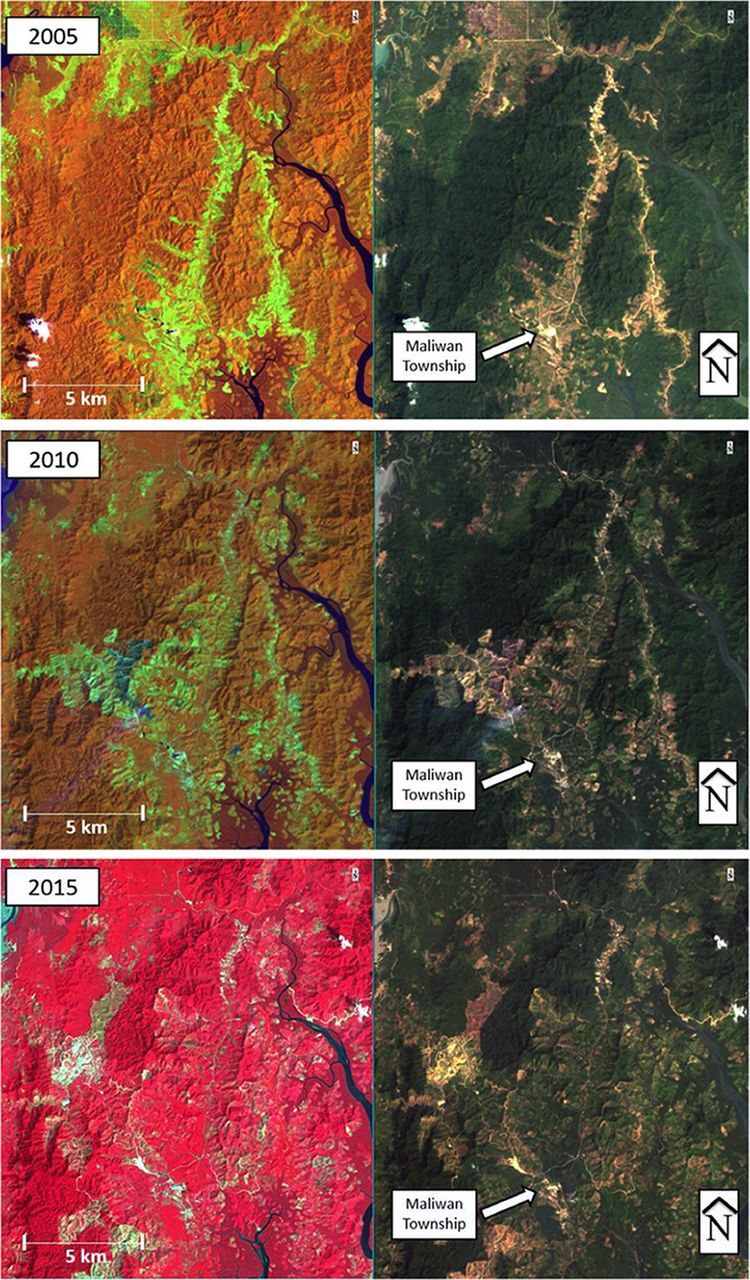
Remote Sensing Vegetation Analysis, 1975–2015 In each set of maps, the rightmost map is the natural color model and the leftmost map is the false color model. 1975: There appears to be little deforestation surrounding Maliwan Township and virtually none in the forested lands to the north. 1990: Surrounding deforestation near Maliwan Township appears to have increased. 2000: A marked increase in deforestation is evident. 2005: Since 2000, the road system has split into 2 main segments and smaller forays into the mountainside branch off of the main road. 2010: The road system is now flanked by cleared land on both sides. 2015: Deforestation has encroached on vast areas of once natural forestland. The road systems have evolved into complex and planned transport infrastructure.

In the Tanintharyi Region, we identified the Maliwan and Kawthaung townships as key regions for oil palm cultivation based on ENVI models. From the 1975 imagery, we see that there is virtually zero observable deforestation near Maliwan township (Figure 3). From the vegetation false-color image, the forested lands in the north are largely identical in hue, indicating no change in forest cover. The area of land directly surrounding Maliwan and the swath of land in the northwest are, however, different in hue. The light pink indicates either poor vegetation or recent deforestation. These results are consistent with historical trends for the Tanintharyi Region.

In 1975, there was virtually no observable deforestration near Maliwan Township.

Next, Landsat imagery from 1990 revealed a slight but noticeable change in vegetation cover compared with 1975 (Figure 3). First, the buffer of cleared land surrounding Maliwan appears to have increased in size. This may be due to growing populations and expanding town boundaries or it may foreshadow the expected intensive cultivation in the region. The northwest region that appeared to be the onset of deforestation in 1975 here finds its parallel. There appears to be little intensive deforestation or road system through the mountain.

Landsat imagery from 2000 revealed a progressive shift in the preceding trends (Figure 3). The first and most striking observation is the beginning of a road system through the mountains. This road is connected to a series of smaller roads around the Maliwan Township, which has also increased in size. The northwestern deforestation observed over the past 20 years progressed well into the western edge of the mountain range. Most important, however, is the bright area of deforestation in the north. This land, ground truthing later revealed, is the beginning of an immense area of land dedicated to intense industrial oil palm cultivation.

Satellite imagery from 2000 revealed a progressive shift in deforestation trends.

Landsat 2005 imagery indicated a progression of this trend (Figure 3). The road system has developed a parallel eastern counterpart that extends further into the hillside and away to the southeast. The Maliwan Township, which was before almost an enclave among the forestland, now connects directly to the primary road system. Additionally, the road system has not only developed an eastern counterpart but also dendrite-like forays into the mountainside. In the vegetation false-color image, recent deforestation, such as that which formed the roads, is a bright yellow. Older deforestation, such as the original swatch of land in the northwest, is now a darker orange, indicating either the return of forest cover or, more likely, the growth of oil palm farms. Lastly, the northern swatch of deforested land looks increasingly like designed, industrial farming. The roads form a grid-like system and deforestation has spread outward.

Next, the 2010 Landsat imagery revealed a continuation and acceleration of the previous trends (Figure 3). First, were it not for the marker indicating the location of Maliwan Township, it might prove difficult to locate its boundaries, because there is little primary forest surrounding it anymore. Furthermore, the primary road system is no longer flanked by forestland, but instead recently cleared land surrounds it. The lighter orange that surrounds the road compared with the pockets of forest adjacent indicates this. There is an additional pocket of recently cleared land immediately northwest of Maliwan, already with a relatively complex road system. Lastly, the industrial oil palm land in the north appears obscured by, in the false-color image, a deep orange, and in the pseudo-natural color image, a deep green. This indicates a high level of vegetation abundance that is most likely intensive crop growth. In this image frame, there appears to be just about as much cleared land now as there is primary forest cover. After only 12 years since the first plantation, this shift is quite significant.

Lastly, the 2015 Landsat imagery depicted the current landscape (Figure 3). Immediately evident in the pseudo-natural color image is the sheer lack of deep green that once dominated the region. The road systems are barely distinguishable, because there is so much deforestation flanking them that it is hard to discern where one road starts and deforested land starts. The infrared image provides very interesting insight here. First, pinks and light reds dominate the spectrum of hues observed. This indicates recent deforestation. The deeper the hue, the more intense the chlorophyll content and vegetation growth. Accordingly, the land with the most vibrant red is, unsurprisingly, the northern edge of the industrial oil palm plantations. This indicates intensive oil palm growth. Given that this image was taken 17 years after the onset of industrial oil palm cultivation in Myanmar, this time scale is on par with the amount of time it takes for planted trees to mature and reach their maximum yield.

### Ground Truthing

Ground truthing validated the observed spatiotemporal land-use changes brought about by the spread of industrial oil palm plantations. In addition to this validation, ground truthing revealed several barriers to oil palm plantation development. First, given the industry's obvious growth, it is curious that fields of abandoned oil palm trees would exist in the region (Figure 4.1). Second, that an entire field of oil palm trees was cut down in favor of another crop in this region is also surprising (Figure 4.2). The healthy adjacent oil palm field in the background indicates that this particular plot of land was not likely fallow or poorly suited to growth. Lastly, separated by a well-paved road winding through the hillside with a golden pagoda in the background, newly planted industrial oil palm trees (as denoted by their short stature) sit across from remaining primary forest cover (Figure 4.3).

**FIGURE 4.**
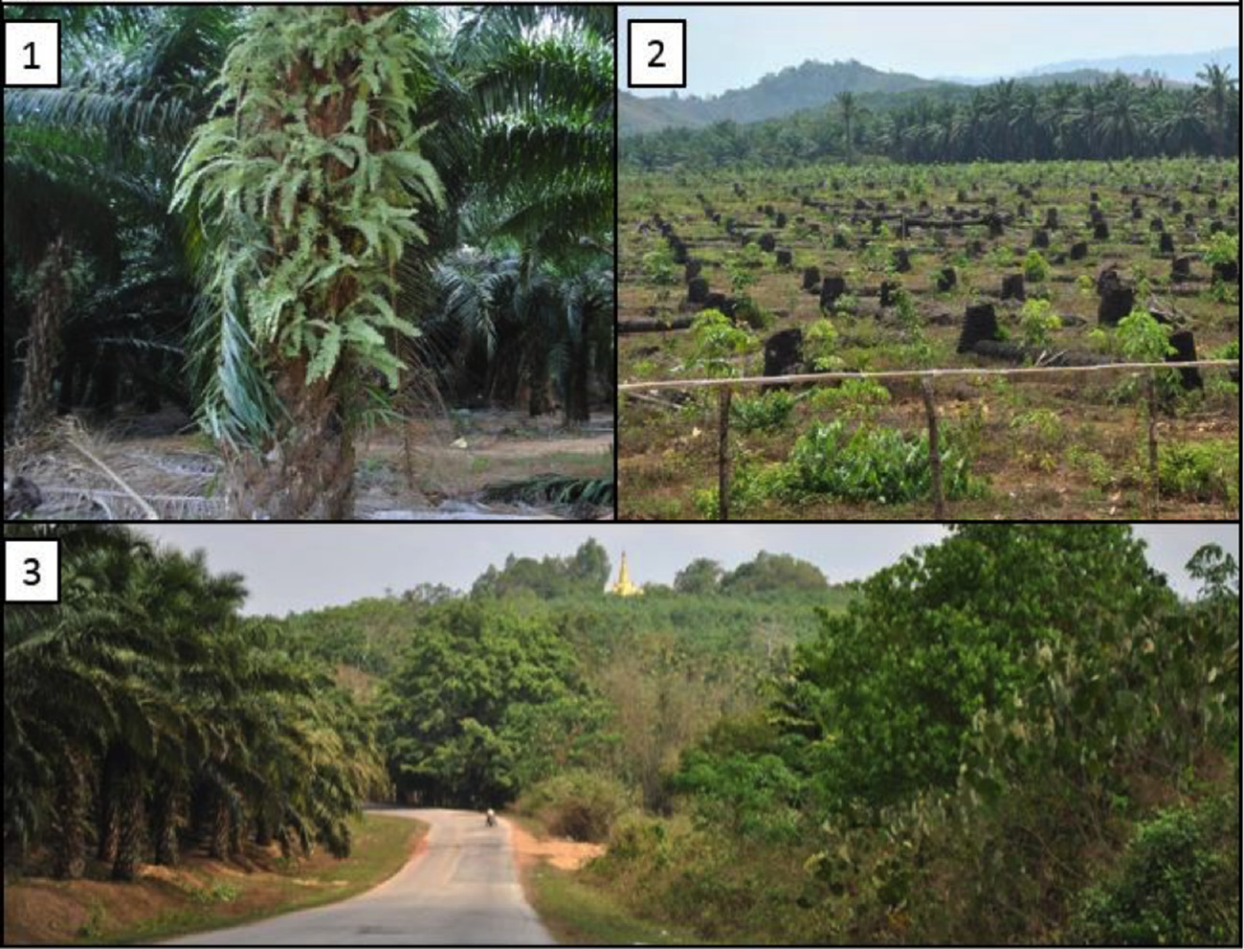
Photo-Based Truth Finding Observations [1] Field of abandoned oil palm trees; [2] Cleared field of oil palm trees with a new, leafy tree species growing in their stead; [3] Paved road flanked on one side by natural tree cover and the other by an encroaching oil palm plantation.

## DISCUSSION

The relationship between planetary and human health within the context of Burmese palm oil can be summarized as follows: as smallholder farming converts to industrial monocultures, deforestation increases and biodiversity decreases. This decrease results in 2 outcomes in Tanintharyi. First, fewer ecosystem services are available for low-income Tanintharyi communities around which plantations develop. Second, extrapolating from this observed development of oil palm plantations in satellite imagery and ground truthing in the context of a government push for edible oil self-sufficiency, palm oil production will increase. This increased production may be concomitant with increased palm oil consumption in low-income communities (assuming increased availability and constant or decreased prices) and an associated increase in health risks within these communities (assuming increased consumption).

As smallholder farming converts to industrial monocultures, deforestation increases and biodiversity decreases.

Increased palm oil production in Myanmar may be concomitant with increased consumption in low-income communities and associated increases in health risks.

However, deforestation in Myanmar need not be necessarily associated with oil palm cultivation, particularly in Tanintharyi. During the reign of Myanmar's military junta, “crony capitalism,” in which business transactions favor affiliates of government and military officials, was rampant.^29^ Logging of Myanmar's natural forestland, especially in Tanintharyi, proceeded unimpeded such that between 1990 and 2010, forest cover in Myanmar fell from 45% to less than 20%.^29^ The Htoo Group, with annual revenues of nearly half a billion dollars, is one of the largest palm oil companies in Myanmar.^30^ Htoo business leader, Tay Za (whose father served with high-ranking military officials), has received massive government land concessions (668,000 acres in Tanintharyi in 2014) and has rapidly logged 162,000 acres allotted his firm as a separate palm oil land concession.^30^ Despite a 2014 ban on timber exports in Myanmar, this raises the question of whether (or perhaps to what extent) logging will still occur, but simply under the guise of oil palm farm development safeguarded under a national edible oil self-sufficiency drive.

Still, despite the impact of illegal (or legally sanctioned, via land concession) logging on the observed deforestation in Tanintharyi, we believe new oil palm plantations will continue to comprise a large portion of the newly deforested lands. In addition to the government stance on edible oil self-sufficiency, we have observed the formation of grid-like plantations (characteristic of oil palm) in Tanintharyi via Landsat data in our remote sensing analyses and have confirmed the existence of these plantations via ground truthing. As such, we maintain that oil palm cultivation in Tanintharyi, if propagated, may impact the diets of low-income communities reliant on its edible oil along with the well-being of surrounding Tanintharyi communities most affected by deforestation. Whether from a planetary or human health perspective, the case for prompt and significant changes in the trajectory of Burmese oil palm growth is strong.

### Implications of This Study

This study confirms that recent oil palm development in Myanmar is concurrent with extensive deforestation and frames this within the context of sustainable diets. These results may have important implications for the future of sustainable diets in Myanmar. To that end, it is important to note that this study focuses on the environmental implications of intensive monoculture oil palm cultivation in rural Myanmar and what downstream health impacts may occur given the described political and economic mediators of palm oil consumption. As we have shown, the extent of deforestation has impacts on the extent and scale of biodiversity loss. This biodiversity loss has negative health outcomes borne particularly by vulnerable, rural populations such as the ones surrounding Maliwan, one site of oil palm cultivation in the Tanintharyi province. However, more research is needed to fully measure the effects of this biodiversity loss on rural diet composition. As we examine the progression of diets and nutrition in Myanmar, it will remain important to contextualize the magnitude and direction of land-use changes in the region given associated biodiversity loss, as outlined in this study. It is also important to note that these impacts of biodiversity represent only 1 of 10 principles of sustainable diets. That is, the food system-based determinants of human health are varied, intertwined, and complex; biodiversity is extremely important, but not of sole importance.

Finally, because low-income populations are the primary consumers of palm oil and live in closest proximity to plantations, they likely bear the brunt of the consequences. If these trends of palm oil intensification continue, 4 key outcomes may unfold: (1) high levels of biodiversity loss, (2) increased accessibility and affordability of edible palm oil, (3) mitigated importation of palm oil domestically, and (4) large profit margins made from selling excess palm oil on the international market. The first 2 implications bode poorly for low-income Burmese people. Higher-income communities are largely unfazed by changing palm oil prices, because they can afford healthier, more nutritious alternatives. The latter 2 implications bode very well for the domestic economy and international players invested in the palm oil market. The Burmese government may view this mitigated reliance on neighboring markets for what can be produced domestically as desirable. Furthermore, given the size of the global palm oil market, it is possible that agrochemical industries with strong interests will have more influence over the government than low-income Burmese communities. Given this distribution of power, we may assume that if the growth of the Burmese palm oil industry proceeds at its current rate and without policy or nutrition interventions, then low-income Burmese communities, especially those in the immediate vicinity of industrial deforestation, may face negative health outcomes for years to come.

## Supplementary Material

17-00132-Nicholas-Supplement.pdf

## Notes

**Peer Reviewed**

**Funding:** Earth Institute, Columbia University.
