# Supplementary material for: Palm Oil in Myanmar: A Spatiotemporal Analysis of the Effects of Industrial Farming on Biodiversity Loss
Source: Glob Health Sci Pract. 2018 Mar 21;6(1):210–22. doi: 10.9745/GHSP-D-17-00132 (PMC5878073; doi:10.9745/GHSP-D-17-00132)
Supplement: 17-00132-Nicholas-Supplement.pdf [file 17-00132-Nicholas-Supplement.pdf]

Nicholas K, Fanzo J, McManus K. Palm oil in Myanmar: a spatiotemporal analysis of the effects of industrial farming on biodiversity loss. Glob Health Sci Pract. 2018;6(1). https://doi.org/10.9745/GHSP-D-17-00132

SUPPLEMENT. Selection of Parameter Thresholds

Drought in Myanmar

While identifying areas of high agricultural growth in Myanmar, we also wanted to consider any limiting factors (besides natural weather parameters) that may affect the growth of the Burmese palm oil industry. Agricultural susceptibility to drought has been a problem in Myanmar for several decades. Specifically, we wanted to understand the spatial distribution of these drought occurrences and if they had any overlap with areas of planned oil palm growth. We gathered geocoded climate data on drought intensity and frequency across Myanmar and layered it with a map of Myanmar showing land zoned for agriculture (Supplement Figure 1). As depicted, there is a strong history of intense droughts in the Tanintharyi region, which we now know as a hotspot for industrial palm oil development, both present and planned.

Supplement Figure 1. Drought Intensity Compared With Agriculture Land Use in Myanmar

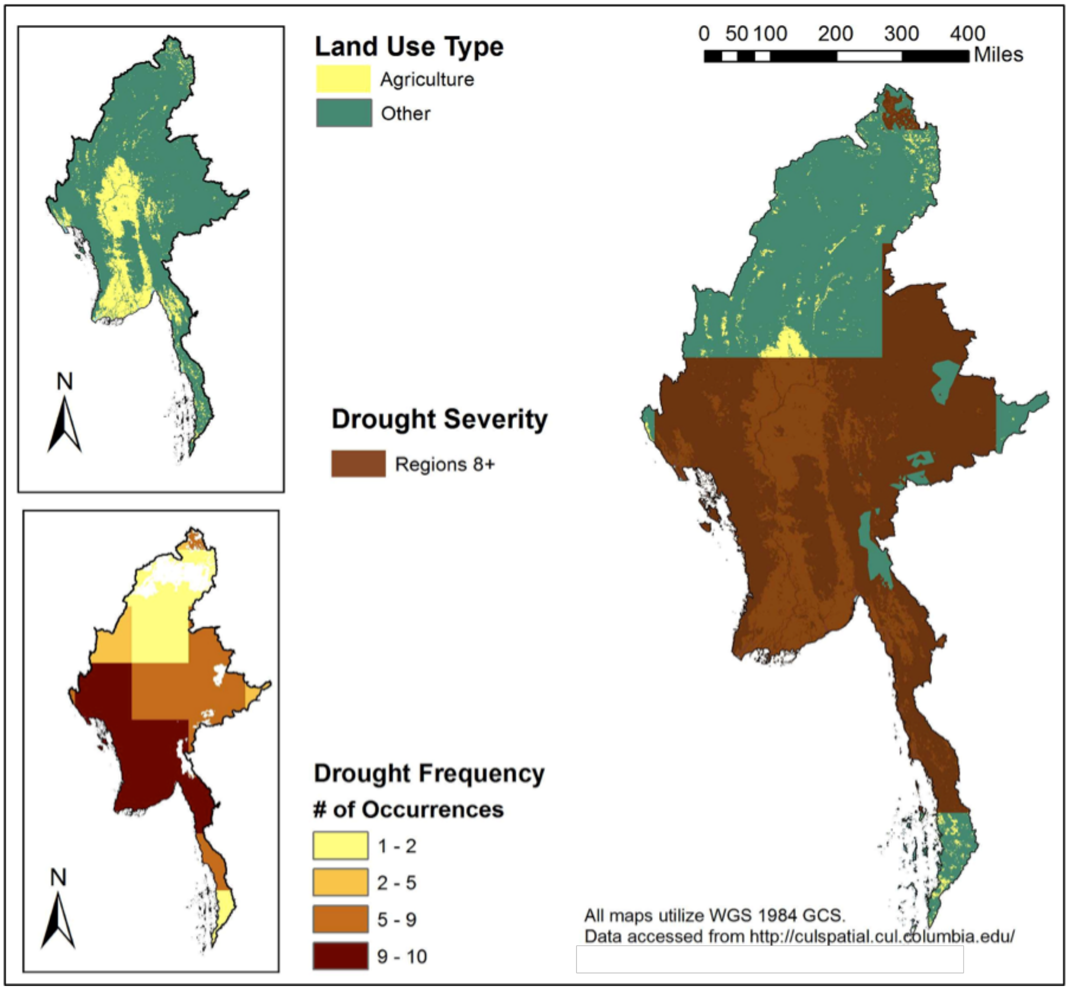

GIS Land Suitability Analysis

In choosing the parameters elevation, rainfall, and slope, we also chose thresholds of each. There are several species of palm oil tree and various locations they are grown. Because there are no publicly available standards for palm oil growth in Myanmar, we conducted a literature review to discern these thresholds. In the end, we based them on two studies conducted in Myanmar and Malaysia.[13,30] Teoh Cheng Hai (2000)[30] advocated for rainfall levels between 1,800 and 2,000 mm/year, a slope gradient no more than 25% steepness, and elevation less than 400 m. Saxon (2014)[13] used rainfall levels between 2,400 and 3,200 mm/year, a slope no more than 16%, and elevation less than 100 m. To identify which parameters were realistic, we ran suitability analyses comparing existing climate data for Myanmar against each parameter condition. The results below explain the logic behind our parameters.

Supplement Figure 2. Rainfall Elevation Comparison

The photo on the left uses Saxon's threshold for rainfall and the right uses Teoh's. Blue in the map represents suitable areas given each cutoff. Comparing their cutoff points to actual climate data in Myanmar shows that Teoh's threshold is too limiting. Accordingly, this study uses Saxon's threshold.

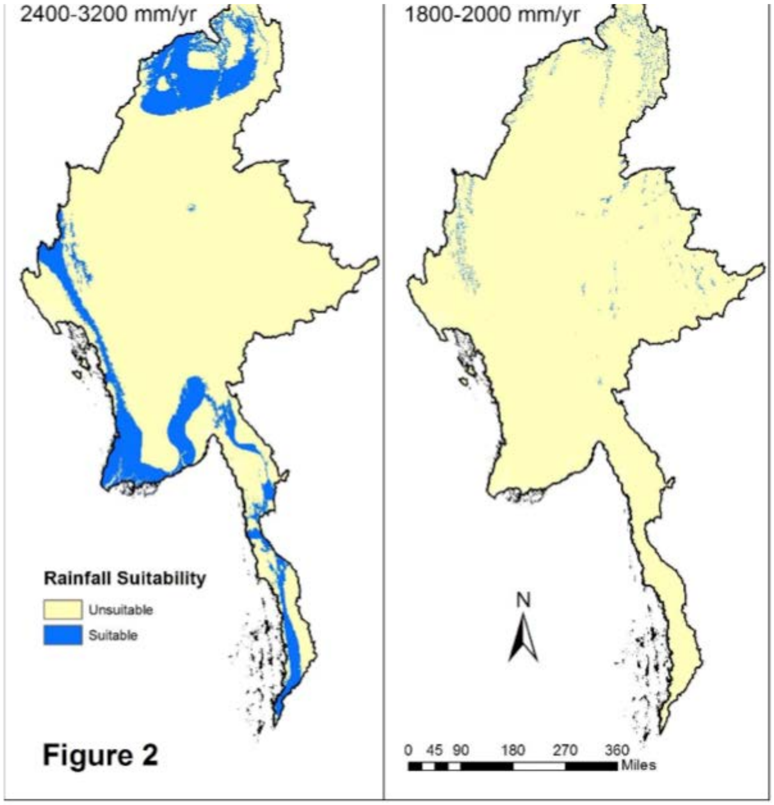

Supplement Figure 3. Slope Suitability Comparison

The green areas represent land that is suitable given each cutoff for slope. Compared with rainfall, the opposite occurs here. There is not enough of a substantial difference to warrant choosing one or the other. This study utilizes the slightly more restricting variable (16%).

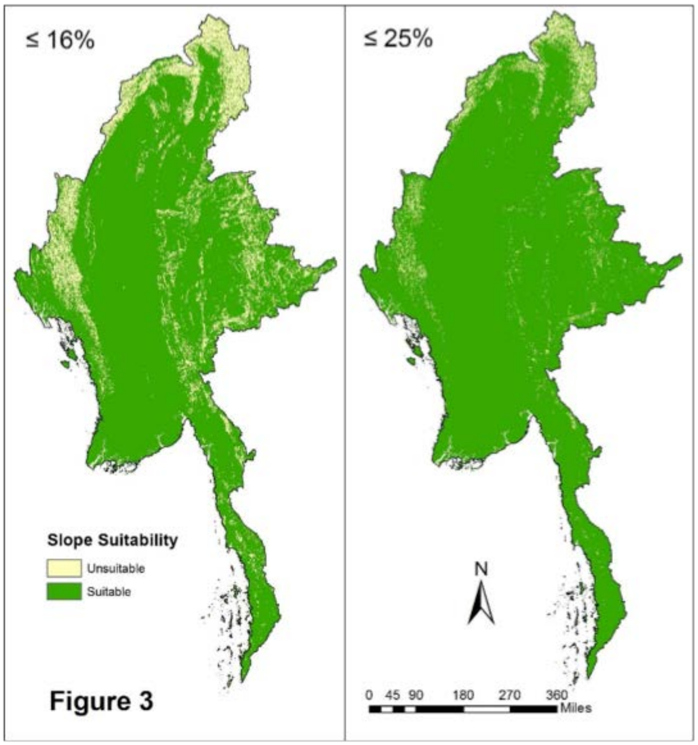

Figure 3
